# Personal reflections on a new role in infectious diseases

**DOI:** 10.4102/sajid.v38i1.583

**Published:** 2023-11-17

**Authors:** Mark F. Cotton

**Affiliations:** 1 Department of Paediatrics Child Health, Faculty of Medicine and Health Sciences, Stellenbosch University, Tygerberg, South Africa

This is my first editorial as editor-in-chief of the *Southern African Journal of Infectious Diseases* (SAJID) since I was appointed in November 2022 and an opportunity for reflection. As someone who first entered the difficult but personally rewarding pathway of writing and publishing in 1988, I am now in at the receiving end. Despite being on the Editorial Board since SAJID’s inception in 2014, I was not very active. Rather, my efforts were towards getting my own manuscripts published, as with most ‘academics’. I receive manuscripts for review and begin the process through an initial evaluation of new manuscripts to ensure the suitability of content for the journal and readability for the reviewers and readers. At this stage, for those not yet suitable for review, if there is potential merit, I offer advice, which in my opinion would strengthen the manuscript and encourage resubmission. If suitable for review, with agreement of the three-person editorial team, we assign responsibility to one of us. The next task is to find suitable reviewers who are knowledgeable in the subject area and willing to review. The process can be lengthy, but seeing a useful article through to publication has immense value for all of us. Our reviews focus on practical advice, aimed at improving the manuscripts and relevance for the readers to deepen understanding. As a clinician, my first publications were case reports, which improved my own clinical insights and gave me experience in the difficult task of writing.^1,2^ Apart from the data presented in a manuscript, language and grammar are key to effectively communicating one’s work. In this era of super-specialisation, it is good to gain a broader understanding of topics. As a paediatrician, I have always enjoyed learning about experience in adults and laboratory aspects of infectious diseases. Much of it applies to children too, either directly or through the families. By representing the Federation of Infectious Diseases of Southern Africa, SAJID covers a broad range of topics highly relevant to where we practice. Topics range from clinical experience, antimicrobial resistance patterns, parasitology, tuberculosis, treatment guidelines and immunisation. Case studies are often a springboard to something new or previously unrecognised. For example, the acquired immunodeficiency syndrome was described clinically almost 2 years prior to the formal identification of the human immunodeficiency virus.^3^ The *Southern African Journal of Infectious Disease* is an appropriate journal for all levels of contributors, from an emerging investigator working on their first submission to leaders in their fields. There is place for all, provided that the manuscripts make meaningful contributions to the field. Many colleagues contribute to making SAJID such a valuable resource. Professor Charles Feldman was editor-in-chief since the journal’s inception in 2014 and oversaw the transition from the Southern African Journal of Epidemiology and Infection (SAJEI), established by Prof. Hendrik Koornhof in 1985 and supported by Priscilla May. Fortunately, Prof. Feldman continues to give guidance as emeritus editor-in-chief. We also thank Prof. John Frean, deputy editor until 2022 and always available for advice as emeritus deputy editor. Professor Andrew Whitelaw is the new deputy editor since November 2022. His expertise and commitment are integral to maintaining standards of excellence. Under Prof. Feldman’s leadership, SAJID is accredited by the Department of Education and Training (DHET) enabling teaching institutions to receive funding for journal outputs. Since 2021, SAJID has been listed on the Directory of Open Access Journals (DOAJ) and indexed on PubMed. A direct consequence of these developments is our first impact factor of 0.9 for 2022. The impact factor is a measure of citations by authors of recent articles from the journal. The reviewers are essential for providing balanced, fair and objective critique. We are extremely grateful to the many reviewers who take time from their extremely busy professional lives to perform this function. All of us on the editorial team know that it is a time-consuming activity – but without reviewers the journal cannot function. With this in mind, we encourage you to accept invitations to review – this serves not only the broader medical and scientific community but also as you undertake more reviews you will improve your own analytical and appraisal abilities. Lastly, we are very grateful to our publisher, the AOSIS team. whose technical expertise is essential for the journal. In conclusion, through all of our collective commitment, SAJID is an invaluable resource for Southern African research and the health of our people. Let us keep it that way and do even better.
